# Synthesis, Characterization and Process Optimization of Bone Whitlockite

**DOI:** 10.3390/nano10091856

**Published:** 2020-09-17

**Authors:** Sadaf Batool, Usman Liaqat, Zakir Hussain, Manzar Sohail

**Affiliations:** 1 School of Chemical and Materials Engineering (SCME), National University of Sciences & Technology (NUST), Sector H-12, 44000 Islamabad, Pakistan; sumar7393@gmail.com (S.B.); usman.liaqat@scme.nust.edu.pk (U.L.); 2 Department of Chemistry, School of Natural Sciences (SNS), National University of Sciences & Technology (NUST), Sector H-12, 44000 Islamabad, Pakistan; manzar.sohail@gmail.com

**Keywords:** whitlockite, bone mineral, calcium phosphate, synthesis, process optimization

## Abstract

Whitlockite, being the second most abundant bio-mineral in living bone, finds huge applications in tissue regeneration and implants and its synthesis into its pure form has remained a challenge. Although precipitation of whitlockite phase has been reported recently in many publications, effects of various parameters to control such phase as well as conditions for the bulk preparation of this extremely important bio-mineral have not been investigated so far. In this work, we report the precipitation of pure whitlockite phase using common precursors. As reported in the literature, whitlockite is stable in a narrow pH range, therefore; optimization of pH for the stabilization of whitlockite phase has been investigated. Additionally, in order to narrow down the optimum conditions for the whitlockite precipitation, effect of temperature and heating conditions has also been studied. The obtained solids were characterized using powder X-ray diffraction (PXRD), Fourier transform infrared spectroscopy (FTIR), Raman spectroscopy, scanning electron microscopy (SEM) and thermogravimetric analysis (TGA). From PXRD analysis, it was observed that heating the precursor’s mixture at 100 °C with subsequent aging at the optimized pH resulted in the precipitation of pure whitlockite phase. These results were further confirmed by TGA, SEM and Raman spectroscopy analysis and it was confirmed that the conditions reported here favor whitlockite precipitation without formation of any secondary phase. These reaction conditions were further confirmed by changing all the parameters like aging, heating time, feed rate of precursors one by one. From PXRD analysis of these samples, it was concluded that not only pH but temperature, heating time, aging time and feed rate effect simultaneously on the precipitation of pure whitlockite phase and a subtle change in any of these parameters could lead to the formation of undesired stable secondary calcium phosphate phases.

## 1. Introduction

Bone is a composite of collagen fibers reinforced with nano-sized apatites i.e., hydroxyapatite (HA) and whitlockite (WH) [1,2]. Hydroxyapatite is the major component of inorganic phase of bone [3], has a hexagonal crystalline structure and maintains bone strength. It was previously considered as the best bone alloplast but its poor solubility and fast degradation under the body’s acidic environment has limited its use. Some recent studies have shown that WH could be the best substitute for hydroxyapatite alloplast. WH has a rhombohedral crystalline structure [4,5], with R3C space group of alkali and alkaline earth metals that exist naturally in the form of Merrillite (Ca_9_MgNa (PO_4_)_7_) [6], β-tricalcium magnesium phosphate (β-TCMP, Ca_2.86_Mg_0.14_ (PO_4_)_2_) Stanfieldite (Ca, Mg)_3_(PO_4_)_2_ [7], β-tricalcium Phosphate (β-TCP, Ca_3_ (PO_4_)_2_) and magnesium or biological WH (Ca_18_Mg_2_H_2_ (PO_4_)_14_/Ca_9_Mg(HPO_4_)(PO_4_)_6_) [8]. Among various forms of WH, β-TCP and WH have been explored biologically since they are highly biocompatible with excellent osteogenic properties. β-TCP was once considered as bone WH due to its close structural resemblance with WH. However, recent powder X-ray diffraction (PXRD) and SEM analysis of bone have revealed that it is WH that exists naturally in the bone while β-TCP is merely a synthetic analog with similar structure [9,10,11]. Due to biological importance, presence of hydrogen and magnesium makes WH different from β-TCP. Magnesium maintains bone metabolism, stimulates growth during earlier stages of bone development and controls the biological functions of the body [12,13]. It exists in the form of octahedral chains of MgO_6_ and PO_4_H in the threefold axis of rhombohedral geometry [14,15] and its deficiency in the body can lead to osteoporosis along with malfunctions of other body parts such as cardiovascular failure. On the other hand, hydrogen helps magnesium in bone growth by camouflaging the immune system as well as gives stability to WH rhombohedral structure [7,16,17,18,19].

Synthesis of biological WH is not easy due to its poor thermodynamic stability and synthesis over a narrow pH and temperature range. For a long time, WH was considered as an intermediate of hydroxyapatite, magnesium brushite and monetite which converts into stable calcium phosphates (CaPs) under ambient conditions and to hydroxyapatite above pH 4.5 [20,21]. Thus, the ability to obtain WH with ideal chemical composition has remained a challenge. Previously, some researchers tried to synthesize WH by incorporating magnesium in hydroxyapatite and in β-TCP crystal lattice but ended up in mixed phases of WH and CaPs [22,23,24,25]. On the other hand, a lot of works have been reported to explore the exact Ca:Mg:P ratios for the synthesis of pure WH phase, however; resulting in mixed phases [26,27,28,29]. Similarly, different CaPs intermediates like monetite, brushite and calcium deficient apatites have also been used as precursors to synthesize pure WH [30,31]. Recently, Magalhaes et al., in 2018, reported that whitlockite phase is stable between narrow pH range of 5 and 6 [8] while in other works acid pH 5.6 and pH 6–7 allows precipitation of β-TCMP and other magnesium containing whitlockite using common precursors [32,33,34] as well as under high-temperature hydrothermal conditions [35] to synthesize pure WH phase. Shah et al., in 2017, did the crystallographic analysis of bone and reported that whitlockite formation in natural system occur in acidic conditions [36] while in another work done by Abdelkader et al. in 2001, basic pH 10 and large magnesium concentration were reported for biological whitlockite precipitation [32]. However, none of these works have reported the exact pH along with all relevant parameters such as heating and aging time for the synthesis of pure biological WH phase resulting in a big question mark on the reproducibility of pure WH phase. Therefore, due to huge potential of this material for biomedical applications, it was extremely essential to explore and report exact conditions for the formation of pure WH phase. Additionally, it was also essential to explore whether the synthesis process is reproducible and can it be optimized for the synthesis of WH at mass scale.

Three batches of reactions were carried out to synthesize homogenous WH i.e., effect of heating conditions and temperature at pH 4 (batch one), effect of temperature and heating time at pH 5 (batch two) and effect of different parameters like aging time, heating time and feed rate on WH precipitation (batch three). Finally, the optimized parameters were checked for the reproducibility of the WH phase as well synthesis of material in grams scale quantity. To the best of our knowledge, we believe, this is the first detailed study on the synthesis of WH phase with comprehensive optimization of various parameters and extending the same for the mass production of this bio-mineral and demonstrates huge commercial value.

## 2. Materials and Method

Orthophosphoric acid was purchased from Honeywell (Fluka, NC, USA), calcium hydroxide from GPR Rectapur and magnesium hydroxide from Duksan (Daejeon, SouthKorea). All the chemicals were used as received without any further processing.

### 2.1. Synthesis of WH

**Figure 1:**
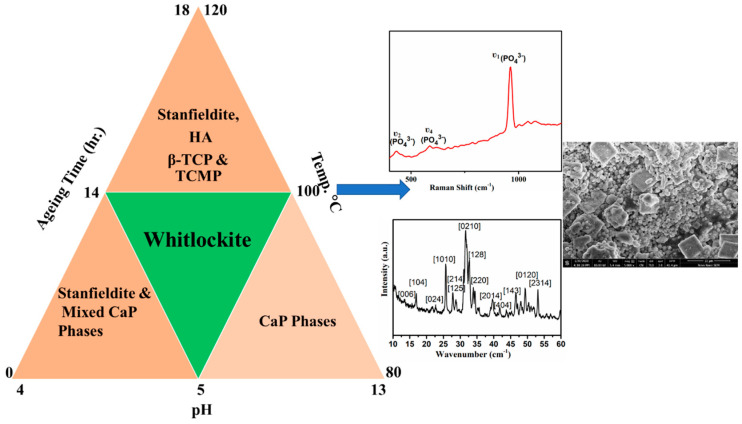
Schematics of approach used for WH synthesis.

Here in, we have reported on the optimization of conditions for the precipitation of WH phase and demonstrated the scale-up capability of our synthesis method. In our method, a wet homogenous chemical precipitation procedure was employed and the effect of different pH conditions on the precipitant dissolution was also examined, starting from pH 13 to pH 1. As more data on acidic pH precipitation were reported therefore, first we added phosphoric acid till pH 1. The precipitates did not form at this pH and already formed precipitates dissolved in reaction mixture. Then we stopped acid addition at pH 2, but the same results as that of pH 1 were obtained. In next reaction, pH 3 was selected as final and it resulted in the formation of turbid liquid. All these samples were kept on aging at room temperature without any agitation for one month but none of the product was obtained. Therefore, we decided to check the stability of precipitates at pH 4 which resulted in formation of stable precipitates of magnesian whitlockite and β-TCMP. As the reported (Ca + Mg/P) ratios were used therefore, we decided to first to check the effect of pH 5 on biological whitlockite formation before changing other parameters. At pH 5, we obtained some precipitation of biological whitlockite in all the three batches while exact conditions were optimized in batch 2. It was observed that below pH 4, the precursor’s precipitates started dissolving with complete dissolution at pH ≤ 2.5 with no reappearance of precipitates even after aging for one month. For the synthesis of WH, Ca:Mg:P ratios were used as reported earlier [20]. Briefly, a 0.37 M solution of Ca(OH)_2_, 0.13 M solution of Mg(OH)_2_ and 0.5 M solution of H_3_PO_4_ were prepared. All the products obtained were filtered, washed thrice with deionized water and dried overnight at 50 °C. All the samples were aged at room temperature without any agitation. Table 1 below shows the overall reaction conditions and formed products besides precipitation of WH phase. The overall approach used for the synthesis of WH is shown in Figure 1.

#### 2.1.1. Synthesis of WH at pH 4-Effect of Heating Conditions

In the first batch, effect of heating conditions/temperature on the synthesis of WH phase at a constant pH of 4 was investigated. Precursor’s solutions were mixed and the mixture was stirred for 30 min at 45 °C followed by the addition of 0.5 M H_3_PO_4_ at 10 mL/5min to the reaction mixture to achieve pH 4. This mixture was divided into three parts as WH-1, WH-2 and WH-3. The WH-1 reaction mixture was kept stirring for 5 h at 80 °C, the WH-2 mixture was autoclaved at 120 °C for 5 h, while WH-3 mixture was refluxed at 100 °C for 5 h. All reaction mixtures were aged for 14 h to achieve the final products.

#### 2.1.2. Synthesis of WH at pH 5-Effect of Heating Conditions

In the second batch, effect of heating conditions/temperature on the synthesis of WH phase at a constant pH of 5 was investigated. In all such cases, addition of phosphate solution was stopped at pH 5. This mixture was divided into three parts as WH-4, WH-5 and WH-6. The WH-4 reaction mixture was kept on stirring for 10 h at 80 °C, the WH-5 mixture was autoclaved at 120 °C for 10 h, while WH-6 mixture was refluxed at 100 °C for 10 h. All reaction mixtures were aged for 14 h to achieve the final products.

#### 2.1.3. Synthesis of WH-Effect of Heating Time, Aging and Annealing

In the third batch, effect of different parameters like heating time, aging, feed rate and annealing on the synthesis of WH phase was studied. In the case of WH-7, heating time was increased to 12 h while in the case of WH-8 aging time was increased to 18 h. In addition, in the case of WH-9, precipitants were separated without aging while in the case of WH-10, feed rate of phosphoric acid addition was changed. A total of 10 mL of phosphoric acid was added without any constant time difference. Finally, the effect of annealing on WH-6 precipitants was studied, and for this purpose the product obtained (WH-6) was annealed at 750 °C for 6 h to achieve WH-11.

### 2.2. Characterization of Materials

For the crystallographic characterization of all prepared solids, Powder X-ray diffractometer of STOE (Darmstadt, Germany) and DRON-8 Bourevestnik (Saint-Petersburg, Russia) were used. FTIR analysis was done using the PerkinElmer, SpectrumTM100 spectrophotometer where KBr pellets containing samples were prepared for the analysis. Raman spectroscopic analysis was done using BWS415-532S-iRaman manufactured by BW TEK INC (Newark, NJ, USA). TGA was done using TGA-5500 Discovery series(TA instruments, New Castle, DE, USA) under a nitrogen environment in the range of 100–1100 °C at a rate of 10 °C/min. The size and shape of the prepared nanoparticles were observed using scanning electron microscopy (SEM), NOVA FEISEM-450(New York, NY, USA).

## 3. Results and Discussion

**Figure 2:**
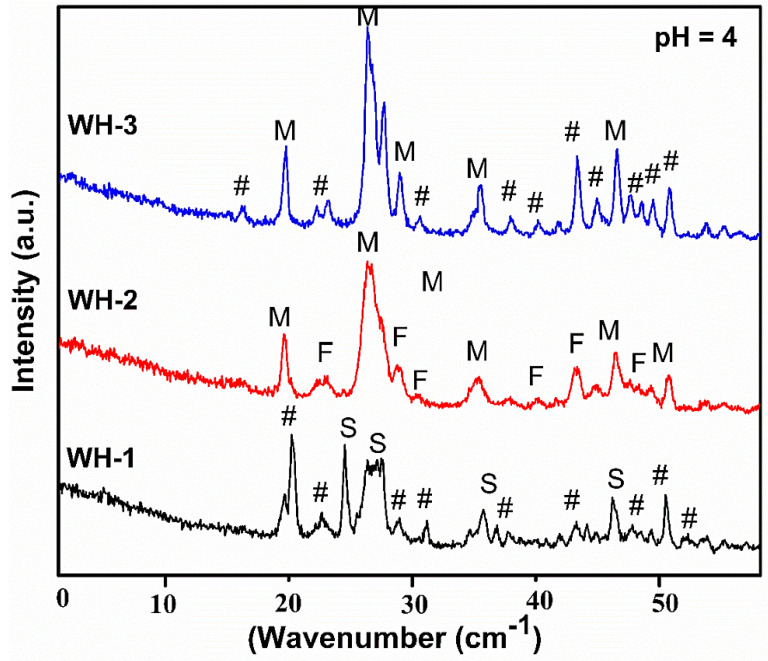
Powder X-ray diffraction (PXRD) diffractograms of the first batch of products (WH-1, WH-2, WH-3) (S for Stanfieldite, F for Farringtonite, M for Monetite and # for mixed CaP phases).

PXRD analysis of the first batch of products (WH-1, WH-2 and WH-3) is shown in Figure 2. Mixed phases of calcium phosphates appeared in XRD diffractogram of WH-1 i.e., calcium pyrophosphate (JCDPS 00-009-0345) [37], magnesium phosphate hydroxide (JCDPS 00-047-0955) [38] and Stanfieldite (magnesian WH) JCDPS 00-013-0404 [39]. The XRD diffractogram of WH-2 shows maximum peaks of Monetite (JCDPS 00-009-0080, 01-070-0360) [40] with some peaks of farringtonite (JCDPS 00-033-0876) and 3rd calcium phosphate oxide (JCDPS 01-070-1379). While, the XRD spectrum of WH-3 does not show any WH phase but peaks of magnesium phosphate (JCDPS 00-0025-1373), Monetite (JCDPS 01-070-1425) [40] and calcium magnesium mixed phosphates (JCDPS 01-082-0503, 00-009-0396). Therefore, PXRD reflections for this group demonstrate that probably pH 4 does not favor the precipitation of WH phase. Although, after the appearance of Stanfieldite peaks in WH-1, we were encouraged to fine tune parameters such as temperature and/or heating conditions to obtain relatively pure WH phase. However, such modifications did not result into a pure WH phase confirming the pH 4 as not desired one to crystallize the required phase.

**Figure 3:**
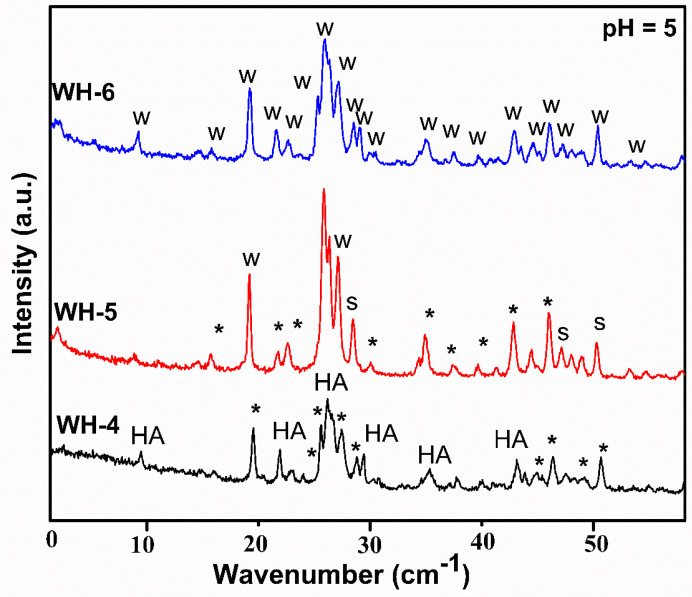
PXRD diffractograms of second batch of products (WH-4, Wh-5, Wh-6) (S for Stanfieldite, W = Biological WH, HA for hydroxyapatite and * for β-TCMP/TCP).

XRD analysis of the second batch of products (WH-4, WH-5 and WH-6) is shown in Figure 3. XRD diffractogram of WH-4 shows the maximum peaks of hydroxyapatite (JCDPS 00-009-0432), β-TCP WH (JCDPS 00-009-0169) [19,41] and some of TCMP (JCDPS 01-077-6692) [26,30]. However, in the XRD diffractogram of WH-5, two peaks of biological WH at 25.9° and 31.4° (JCDPS 01-070-2064) are shown. The other peaks at 46.7° and 49.4°, 42.2° are of TCMP (JCDPS 01-083-1888) while peaks at 51.2° and 56.6° are due to the presence of the Stanfieldite phase (JCDPS 00-013-0404) [8,26]. Additionally, presence of few other peaks represents, calcium and magnesium phosphates mixed phases. In the case of WH-6, precipitation of pure biological WH (C_18_H_2_Mg_2_(PO_4_)_14_) matched with literature [20,30,42,43] as well as JCDPS (01-070-2064 and 00-042-0578). The XRD reflections of WH-4 and WH-5 demonstrated the precipitation of mixed WH phases of Stanfieldite, TCMP and β-TCP which gave evidence that WH precipitation could be possible at pH 5 while a slight change in heating conditions could give pure WH phase. The XRD reflections of WH-6 confirmed this assumption. However, the PXRD analysis of WH-5 also demonstrated that temperature above 100 °C did not favor the formation of pure WH phase. Since magnesium replaces calcium ions completely in the WH crystal lattice, it could lead to the formation of slightly distorted structure which is unstable under high pressure and temperature conditions (120 °C). Moreover, in order to further check the purity of WH-6, high-temperature treatment was carried out where PXRD results confirmed WH-6 as pure biological WH phase. If WH-6 has any secondary amorphous phases, it would be converted to other more stable crystalline phases upon heat treatment [20]. Another evidence of the formation of pure WH phase could be associated with the presence of 0210 peak in WH-6 and WH-11 (discussed later), a characteristic peak of WH. The reduction in *d*-spacing values (from 2.60 to 2.58 at 34.7°) of WH-6 and WH-11 also confirmed the precipitation of pure WH phase [1,44,45,46]. Moreover, the absence of peaks at the 001 face and at 31.2° and 31.8° of β-TCP and hydroxyapatite have demonstrated another evidence of the purity of WH-6 phase [47].

**Figure 4:**
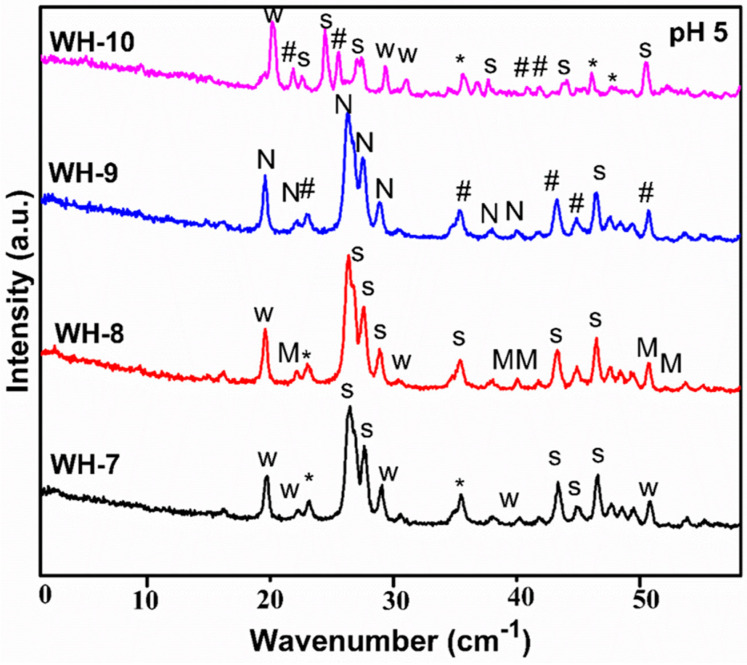
PXRD diffractograms of third batch of products (WH-7, WH-8, WH-9, WH-10) (S for Stanfieldite, W = Biological WH, M for Monetite, N for Newberyite, # for mixed CaP phases and * for β-TCMP/TCP).

PXRD analysis of the third batch of products (WH-7, WH-8 and WH-9, WH-10) is shown in Figure 4. This batch of experiments was carried out to confirm whether the synthesis conditions of WH-6 are ideal for the precipitation of pure WH phase. In the case of XRD diffractogram of WH-7, mixed phases of Mg WH i.e., Stanfieldite, β-TCMP (JCDPS 00-042-0578, 01-077-0692 and 01-070-2064) are shown. Similarly, the XRD spectrum of WH-8 showed minor peaks of WH at 26.3°, 36.0° and 41.0° while other peaks are a mixture of Stanfieldite [48] as well as of Monetite (JCDPS 01-077-0692, 01-070-1425 and 00-042-0578). However, XRD diffractogram of WH-9 did not show any peak of WH phase, but instead peaks of Newberyite [49] (JCDPS -00-035-0780) and calcium phosphate (JCDPS 01-082-0807, 00-009-0169) could be seen, confirming aging as one of the important factors for the precipitation of WH phase along with pH. Furthermore, XRD diffractogram of WH-10 showed peaks of WH at 22.4°, 36.1°, 37.4° (JCDP 00-042-0578) while the rest of the peaks corresponded to Stanfieldite, β-TCP and calcium magnesium phosphate (JCDPS 01-072-2042, 00-012-0404, 00-09-0169, 00-020-0348). These results have also demonstrated the precipitation of pure WH phase which was favored by the slow addition of phosphate precursor.

**Figure 5:**
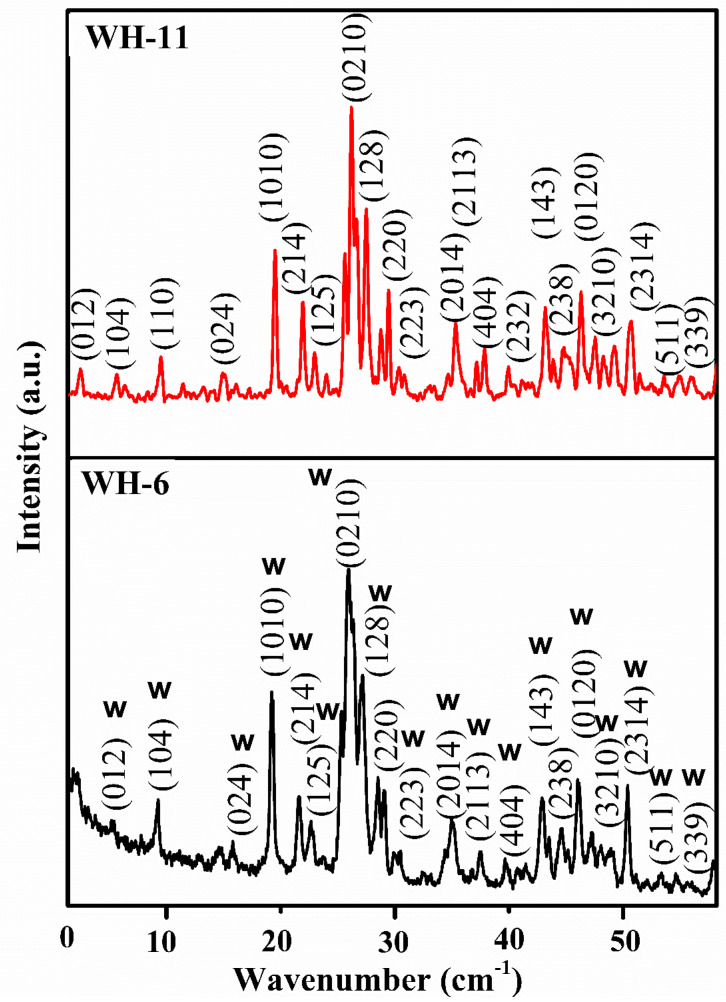
PXRD diffractograms of third batch of product (WH-11) through annealing of WH-6.

Finally, in order to confirm the phase purity of WH-6 sample, it was further annealed to obtain WH-11 (Figure 5). XRD diffractogram of WH-11 showed no significant change in peak position and appearance of no new peak further confirmed WH-6 phase for pure biological WH. Presence of the 0210 peak as the characteristic peak of pure WH phase in WH-6 and WH-11 has also demonstrated the phase purity under given conditions. Therefore, XRD analysis of the third batch of products has demonstrated that pure WH phase can only be precipitated out in a narrow range of temperature and pH while aging can also be considered as one of the essential limiting factors for the precipitation of pure WH phase.

XRD results have clearly demonstrated the formation of pure WH phase in the case of WH-6. Therefore, this product was further characterized for chemical composition as well as morphological investigation.

**Figure 6:**
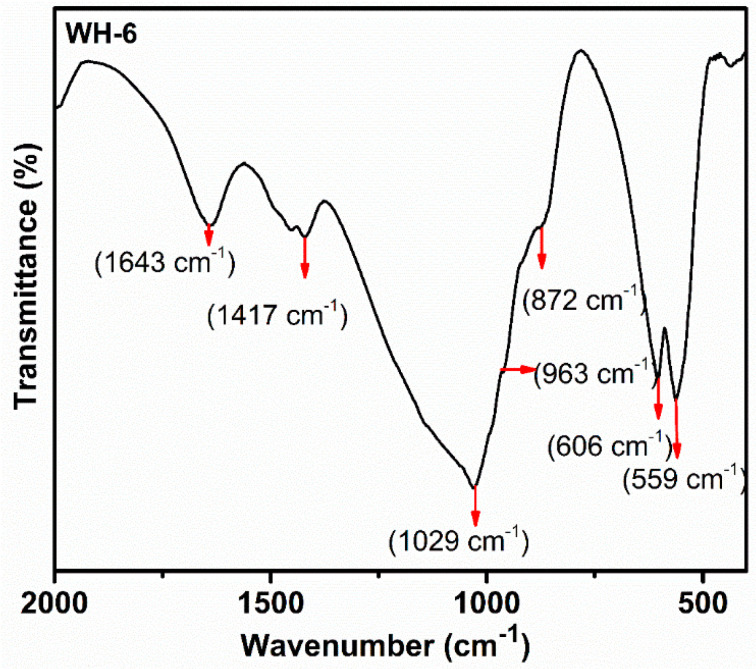
FTIR spectrum of WH-6 product (pure WH phase).

The FTIR analysis (Figure 6) of WH-6 showed a peak at 1029 cm^−1^ which is the peak of υ_3_ phosphate group (PO_4_^3−^). Peaks at 963 and 606 cm^−1^ could be associated with the stretching vibration (υ_1_) of the O-P-O bond and the bending vibration (υ_4_) of the O-P-O bond of phosphate (PO_4_^3−^) group. The asymmetric stretching band of (PO_4_^3−^) group can be observed at 1120 cm^−1^. The peak at 872 cm^−1^ is the characteristic for HPO_4_^2−^ group [20,50]. The XRD results were verified by the FTIR analysis of the WH-6 product. The presence of a peak at 872 cm^−1^ confirms the presence of HPO_4_^2−^ which is the only peak that differs in biological WH from the rest of the WH [20,45]. Moreover, the absence of peaks at 650 cm^−1^ and 3570 cm^−1^ confirmed that our samples were free from the secondary phase [7,31,43]. The peaks below 500 cm^−1^ are also present in the literature and are unidentified [35,51]. The main FTIR region used for whitlockite and other CaPs is between 800 cm^−1^ to 1100 cm^−1^ wavelength. Therefore, only this region is more focused. Researchers have not considered this region [20,43].

FTIR results were also verified by Raman spectroscopic analysis as it gives more insight about magnesium addition into the WH phase as compared to the XRD.

**Figure 7:**
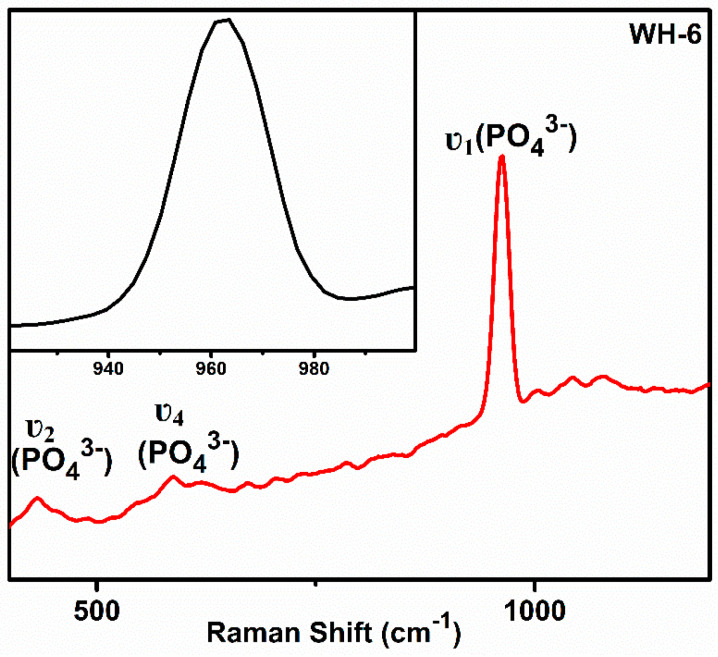
Raman spectrum of WH-6 product.

The Raman spectroscopic analysis of the WH-6 product (Figure 7) showed specific peaks at 962 cm^−1^ and 586 cm^−1^ which are the characteristic peaks of υ_1_ and υ_4_ PO_4_^3−^ ion while the peak at 432 cm^−1^ is a characteristic peak of υ_2_ PO_4_^3−^ [29]. Therefore, both Raman as well as FTIR results have clearly demonstrated the presence of WH phase in WH-6. The Raman spectrum of bone apatite showed characteristic peaks in 959–975 cm^−1^ range and at 432 and 578cm^−1^ for υ_2_ and υ_4_ PO_4_^3−^. It also showed peak broadening in the 959–975 cm^−1^ region due to a shift in PO_4_^3−^ modes which occur as a result of the deformation of PO_4_^3−^ ionic structure by surrounding magnesium ions. The Raman spectrum of hydroxy apatite and β-TCP showed sharp peaks in this region and a broadened peak at 960 cm^−1^, confirming the biological WH [29,36,52].

**Figure 8:**
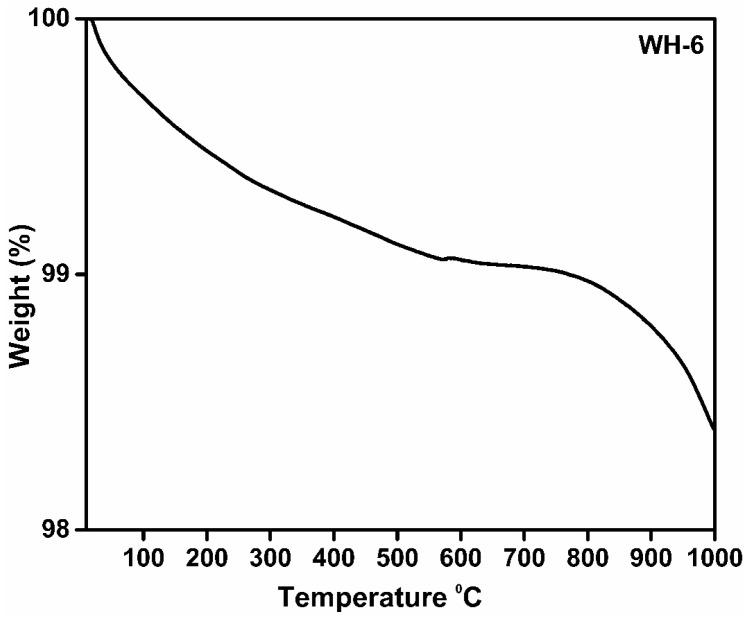
TGA graph of WH-6 product.

In order to study the degradation behavior/phase purity of WH-6, its TGA was carried out. The TGA of WH-6 product (Figure 8) showed a continuous weight loss till 550 °C which could be due to the adsorbed water in crystal lattice of solid. Furthermore, a slight transition is also observed after 550 °C which could be due to breakage of Mg-HPO_4_^2−^ bond. A sudden weight loss has been observed after 700 °C to 1000 °C which represents the conversion of WH into β-TCP and other magnesium phosphates. Previous research work showed that TCP becomes stable up to 1400 °C by the addition of magnesium in the crystal lattice and results in the formation of TCMP and highly stable β-TCP [22,25]. However, our synthesized WH-6 sample degraded at 1000 °C which confirms that magnesium is subsumed completely into the CaP crystal lattice at C5 and C4 position, forming (Ca_18_Mg_2_(HPO_4_)_2_(PO_4_)_12_) rhombohedral structure. Since ionic radii of magnesium ions are smaller than calcium, crystalline stability at a higher temperature is decreased. Moreover, the weight loss after 700 °C could be attributed to the change of HPO_4_^2−^ into P_2_O_7_ and PO_4_ and a rapid weight loss after 900 °C could be associated with a unique phenomenon of WH since it converts into TCMP and β-TCP after 700 °C [20,53,54]. The TGA results of WH-6 matches exactly with the previous literature [18,45,46].

**Figure 9:**
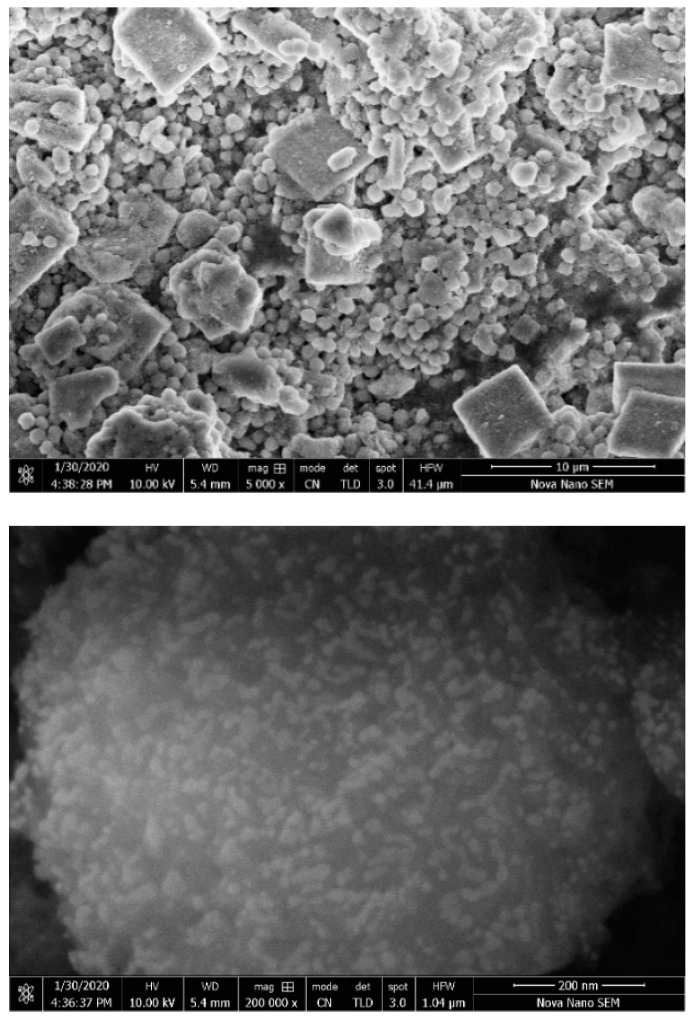
SEM images of WH-6 product with rhombohedral crystals.

Finally, in order to visualize the surface morphology as well as particle size of the WH phase, scanning electron microscopic images of the prepared WH-6 product were obtained. Figure 9 shows the growth of rhombohedral microspheres with nanoparticles grown on their surfaces, similar to the bone nodules reported in the literature [11,18,36,42].

Finally, in order to investigate the reproducibility of the precipitation process to produce pure WH under the optimized conditions, reaction was repeated with larger quantities of the precursors. In this case, initially mixed phases of WH and CaPs appeared, which after annealing for 6 h at 750 °C resulted into pure WH phase, confirmed again through the characterization techniques available. Moreover, in a single reaction with a final reaction volume of 500 mL, we could achieve 25 g of the pure WH. Obtained product have clearly demonstrated the proof of concept for the bulk preparation of pure WH under the optimized conditions. Preparation of pure WH in grams quantities have clearly demonstrated its potential for commercial applications where HA is currently being explored.

## 4. Conclusions

The optimum conditions for precipitation of pure biological WH phase are reported. WH is second abundant inorganic phase of bone having properties superior then that of hydroxyapatite (HA) and β-tricalcium phosphate (TCP). Synthesis of WH phase is not much explored and WH synthesis remained a challenge due to narrow range of parameters for WH stability. From our given data, it is concluded that there are several parameters which influence simultaneously on pure WH phase precipitation. pH is the most important parameter along with temperature and aging. Without aging no WH phase appears making it second most important parameter for WH precipitation. Other parameters such as feed rate, temperature and heating time also influence on the separation of pure phase. Slow addition and short time heating are favorable since magnesium replaces calcium in WH phase and the crystal structure produced is slightly distorted which probably could not maintain stability over long heating periods as well as slow addition favors magnesium to substitute calcium site completely. This work has great scientific importance since grams quantities of the pure WH phase can be produced under the optimized conditions and such material could help to imitate the exact inorganic phase of the bone containing all the essential minerals.

## Figures and Tables

**Table 1:** Effect of various reaction parameters on whitlockite (WH) synthesis (WH-1 to WH-11).

Sr. #	Sample Name	pH	Reaction Conditions	Aging Time	Product
1.	WH-1	4	80 °C/5 h/stirring	14 h	Ca pyrophosphate, Mg phosphate hydroxide, Stanfieldite (Mg WH)
2.	WH-2	4	Autoclaved/120 °C/5 h	14 h	Monetite, farringtonite (WH Phase), Ca phosphate oxide
3.	WH-3	4	Refluxed/100 °C/5 h	14 h	Mg phosphate, Monetite, Ca-Mg mixed phosphates
4.	WH-4	5	80 °C/10 h/stirring	14 h	HA, β-TCP (WH Phase), β-TCMP (WH Phase)
5.	WH-5	5	Autoclaved/120 °C/10 h	14 h	Bone WH, Stanfieldite (WH Phase), β-TCMP (WH Phase)
6.	WH-6	5	Refluxed/100 °C/10 h	14 h	Bone WH
7.	WH-7	5	Refluxed/100 °C/12 h	14 h	Stanfieldite, β-TCMP (WH-Phase)
8.	WH-8	5	Refluxed/100 °C/10 h	18 h	Bone WH, Stanfieldite (WH-Phase), Monetite
9.	WH-9	5	Refluxed/100 °C/10 h	No aging	Newberyite, Ca Phosphate
10.	WH-10	5	Orthophosphoric acid feed rate changed/refluxed/100 °C	14 h	Bone WH, Stanfieldite, β-TCP and Ca-Mg phosphate
11.	WH-11	5	WH-6/annealed/750 °C/6 h		Bone WH
